# Planned Relocation and Health: A Case Study from Fiji

**DOI:** 10.3390/ijerph18084355

**Published:** 2021-04-20

**Authors:** Celia McMichael, Teresia Powell

**Affiliations:** 1 School of Geography, Earth and Atmospheric Sciences, University of Melbourne, Parkville, VIC 3010, Australia; 2 Pacific Centre for Environment and Sustainable Development (PACE-SD), Marine Campus, University of the South Pacific, Suva, Fiji; teresia.powell@usp.ac.fj

**Keywords:** climate change, planned relocation, health, qualitative research, Fiji

## Abstract

In Fiji, low-lying coastal villages are beginning to retreat and relocate in response to coastal erosion, flooding and saltwater intrusion. Planned relocation is considered a last resort as a form of adaptation to the impacts of climatic and environmental change. The health impacts of planned relocation are poorly understood. This paper draws on data from multi-year research with residents of the iTaukei (Indigenous) Fijian village of Vunidogoloa. We used qualitative research methods to examine experiences of planned relocation, including residents’ accounts of their health and quality of life. In-depth interviews and group discussions were conducted with villagers living in a site of relocation, at four points in time (2015, 2016, 2019, and 2020). Twenty-seven people in Vunidogoloa, Fiji, participated in in-depth interviews, several on more than one occasion. Six group discussions with between eight to twelve participants were also conducted. Qualitative analytic software (NVivo) was used to analyse interview transcripts and identify themes. Villagers report both health benefits and challenges following planned relocation. Key facilitators for good health include movement away from some environmental risks to health, adequate drinking water and sanitation, food security including through farms and kitchen gardens, livelihood opportunities, improved access to schools and health services, and appropriate housing design. However, residents also refer to unanticipated risks to health including increased consumption of packaged goods and alcohol, disruptions to social structures and traditional values, and disrupted place attachment following movement away from a coastal site of belonging with consequences for mental wellbeing. Therefore, planned relocation has altered the social determinants of health in complex ways, bringing both health opportunities and risks. These results highlight the need for context-specific planning and adaptation programs that include meaningful involvement of community members in ongoing decision making, and call for an understanding of diverse social determinants of health that emerge and evolve in contexts of planned relocation.

## 1. Introduction

Parallel frontiers of research investigate the implications of climate change for both human health and human mobility. The first of these frontiers demonstrates that climate change is a health risk amplifier through increasing exposure to extreme weather conditions, thermal extremes, altered food yields, water insecurity, foodborne and waterborne disease, changes in air quality, and changes in the geographic range of disease vectors and transmission [1,2,3]. The second of these frontiers suggests that climate change will influence human mobility as people move from sites of climate-related risk; this mobility is variously understood as a crisis to be managed or a form of adaptation to climate risks [4]. Few empirical studies, however, have examined the nexus between climate change, human mobility and health [5].

This paper focuses on planned relocation, which refers to organised movement of people and settlements, typically with the support of a partner agency and/or the state [6]. Planned relocation has been positioned as an adaptation response to sea-level rise and associated impacts for low-lying coastal communities, including in small island nations [7,8]. To date, there are few examples of planned relocation that are precipitated by climate impacts; so, limited empirical research focuses on this topic. No research around climate-related planned relocation focuses specifically on health risks and opportunities [9].

Health is central to human development. It can provide a key indicator of whether planned relocation is a successful and sustainable adaptive responses to the impacts of environmental and climatic change. Research into the (debatably) analogous examples of development-forced displacement and resettlement (e.g., in response to large-scale hydropower schemes) highlights the substantial risks for human development and the determinants of health, including food insecurity, unhealthy water and sanitation system, lack of housing, lack of land, unemployment, homelessness, marginalization, and loss of access to shared assets [10].

Planned relocation in contexts of climatic and environmental change will also affect the social determinants of health. To clarify, social determinants of health are the ‘conditions in which people are born, grow, work, live, and age…[which] are shaped by the distribution of money, power and resources at global, national and local levels’ [11]. For people and populations that relocate to new sites, these conditions can change, with consequences for health, including for example: living conditions, livelihoods and employment, familial and social networks, food environments, gender relationships, built environments and infrastructure, water and sanitation standards, healthcare and education access, legal status, governance, socio-political contexts, ecosystem goods and services, and the aesthetic and cultural benefits of landscape and place [12,13]. An appreciation for the social determinants of health is required, that can look beyond the environmental and climatic drivers of planned relocation, and the focus on relocation as an adaptive strategy that enables people to move out of harm’s way [13,14].

This article focuses on the health opportunities and challenges of planned relocation of a low-lying village in Fiji. It reports the results of analyses of qualitative interviews and group discussions conducted with residents of Vunidogoloa, a small low-lying coastal village that relocated to higher land in 2014. Qualitative research is increasingly used in environmental health research, and has been positioned as a method that can complement or inform exposure assessment and epidemiologic studies [15,16]. We analyse qualitative data to examine people’s perceptions of the health determinants and impacts of planned relocation. We place these experiences into a broader context of climate adaptation and environmental justice.

## 2. Context and Methods

### 2.1. Vunidogoloa, Fiji: The Context

Fiji is a small island developing state and is among the countries most vulnerable to climate change impacts. It has a population of 884,887 people and comprises 322 islands. In 2000, 133,323 people were estimated to live in Fiji’s Low Elevation Coastal Zone (LECZ) area (the LECZ is commonly defined as the zone of land along the coast and below 10 metres of elevation). Because almost all of Fiji’s 300 inhabited islands are at least moderately hilly, planned relocation (as a last resort) from vulnerable areas is feasible without need for international migration [17].

The Fiji Government issued planned relocation guidelines in 2018, which outline guiding principles and procedures for Fijian government, donors and other stakeholders, and communities [18]. Planned relocation is defined as ‘a solution-oriented measure, involving the State, in which a community is physically moved to another location and resettled permanently there’ [18]. The guidelines state that relocation must ensure access to basic human rights including the right to: water, food, health, work, education and a clean and healthy environment [18].

This paper focuses on one research site: Vunidogoloa village, Cakaudrove Province. This village is well known in Fiji as the first village to relocate with government support away from a site of environmental risk. It has been the subject of numerous media reports in Fiji and internationally. It was selected as a research site based on media reports, policy documents, expertise of one of the authors (Teresia Powell), and the advice of other stakeholders in Fiji.

Vunidogoloa is a small *iTaukei* (Indigenous) village from Cakaudrove Province that moved to a higher site, two kilometres inland albeit within *mataqali* (clan) land. The old coastal village (26 households, population ∼140) experienced worsening flooding, erosion, saltwater intrusion, and seawall failures. Earlier adaptation efforts included repair and retreat of some houses and construction of two seawalls. In 2006, Vunidogoloa’s residents approached the Government of Fiji seeking financial assistance for relocation. Other socioeconomic factors may have contributed to villagers’ decision to relocate, including the distance from the road and transport, and limited access to services [19].

The entire village relocated in January 2014 [19,20]. Many ministries and donor agencies (e.g., International Labour Organization) contributed funding and resources; community members contributed labour, building materials, catering, and funds gained through logging ancestral land. A logging license was issued by the Government of Fiji, with logging handled by a local company (i.e., Vitiana Timber Limited). The new village has four fishponds that produce trout, pineapple and banana plantations, a copra drier, and farms. Thirty timber-framed houses were built to consistent specifications, as well as a community hall. However, infrastructure works are not complete (e.g., waste-water drainage). At the time of final data collection (2020), villagers were awaiting a second phase to complete infrastructure works. They raised further funds through logging, and community members were completing the construction of a church at the new site. 

### 2.2. Methods, Recruitment and Analysis

This longitudinal case study employed qualitative research methods. Fieldwork was conducted in Vunidogoloa over a five-year period: November–December 2015, June 2016, May 2019, and March 2020. 

Qualitative case studies are commonly used in the social sciences to provide in-depth answers to questions of ‘how’ and ‘why’; they address research requiring context-dependent analysis. Qualitative longitudinal methods are particularly appropriate to this study because, first, quantitative measurement of changes in health before and after relocation is difficult in small communities with minimal health reporting systems. Second, qualitative methods are of value to environmental health research as they can examine local perspectives, understanding and action on the environmental and social determinants of health [16].

The data derive from: *talanoa* discussions (the iTaukei word *talanoa* derives from tala which means talking or telling stories, and noa which means without concealment); qualitative interviews with individuals and small groups; and observation of the built and ‘natural’ environment and everyday activities. We conducted *talanoa* in the community hall or the home of the Turaga ni Koro (village head) and interviews were conducted in people’s homes or while walking around the village grounds and *mataqali* land. 

This research focused on individuals who relocated from the coastal village site to the new site, and who were ≥18 years of age at time of data collection. We consulted with village leaders to ensure that people of different ages and gender, and with potentially diverse experiences of relocation, were included in the research. Given the small size of the village, and the longitudinal methods that allowed establishment of trust between researchers and residents, we were able to include a diverse and comprehensive sample of community members. There may have been an under-representation of those residents who travel for work and study and so were absent during points of data collection. Local custom shaped attendance at the *talanoa* with the *Turaga ni Koro* calling villagers to discussions. This recruitment method was appropriate to the sociocultural context. Villagers’ participation in data collection was enabled through the involvement of two iTaukei Fijian researchers, Teresia Powell (author) and Manasa Katonivualiku (deceased). 

Interviews followed a semi-structured interview theme guide. We explained research aims to potential participants and then obtained verbal informed consent to participate. The research examined experiences of local environmental change and planned relocation, including the perceived health impacts of planned relocation. Interviews and *talanoa* included discussion of social determinants of health including environmental risk, housing, livelihood opportunities, social capital, cultural identity and traditional knowledge, land security, access to traditional fishing grounds and subsistence farmland, diet, and access to health services. 

Interviews and *talanoa* were conducted in English or in local Fijian dialects with translation provided by local research counterparts. They lasted from 25 min to two hours. They were audio-recorded and transcribed verbatim or recorded as written notes and reviewed for accuracy. 

Data validity was increased through triangulation. Within this qualitative research, triangulation did not aim to confirm and verify the accuracy of specific findings through multiple methods, but rather to produce a deeper understanding of the complex phenomenon of relocation. In our research, data triangulation was achieved through interviews conducted with individual and groups of residents at multiple points in time, *talanoa,* informal ‘between-methods’ discussion with residents, and interactive mobile interviews that allowed a combination of observation, discussion and place-based conversation as participants showed us around their current village, old village, farmland and *mataqali* land. Emerging topics informed subsequent data collection; this iterative process allowed us to identify key themes, note topics where further information and insight would be of value, and identify areas for ongoing enquiry [21].

These data were analysed using thematic content analysis. At each data collection time-point researchers discussed the emergent themes using content analysis to establish consistency and agreement in our conceptual understanding and organization of data. Longitudinal data collection provided opportunity to discuss and confirm themes and earlier findings with participants, allowing iterative analysis. We conducted both synchronic analysis (cross sectional, after each data collection timepoint) and diachronic analysis (longitudinal, using data from multiple timepoints). NVivo qualitative data analysis software was used to code data, and identify final themes and representative quotes [21,22]. We referred to the transcripts to ensure that interpretations of statements were consistent with participants’ intended meaning.

Research permits were granted by the Government of Fiji, and research approvals was provided by the Cakaudrove Provincial Councils and the *Turaga ni Koro* (village headman). Human Research Ethics approval was granted by The University of Melbourne.

## 3. Results: Planned Relocation and the Social Determinants of Health 

In Vunidogoloa, 27 people (14 men, 13 women) participated in interviews. Four people participated in interviews at more than one time-point. In addition, six *talanoa* with between eight to twelve participants were conducted: 2015 (1 *talanoa*), 2016 (2), 2019 (2), 2020 (1). Participants in interviews and *talanoa* ranged in age from early 20s to 84 years. All participants were iTaukei, reflecting the Indigenous ethnicity of the village. The results are presented according to themes identified in interviews and *talanoa* regarding the health benefits and risks of planned relocation (see Table 1).

### 3.1. Environmental Change

Residents of Vunidogoloa are aware of climate change. They refer to environmental changes they experienced at the ‘old village’ site that they attribute to climate change, including sea-level rise, coastal erosion, coastal flooding, saltwater intrusion and associated damage to crops, destruction of sea-walls, loss of coconut trees, and damage to homes from flooding. Villagers suggested that the high tide level rose rapidly in the previous ten to fifteen years, although coastal changes were said to be occurring as early as the 1960s. Residents suggested that these coastal changes did not lead to substantial health risks at the old site: spring water was supplied by gravity-fed pipes such that there were not concerns around saltwater intrusion of aquifers; and there was no reported increases in mosquito- (e.g., dengue) or waterborne diseases (e.g., from cholera-carrying copepods that live in salty or brackish water). 

Yet, several people referred to health risks at the old site that they linked to coastal flooding. A few suggested that a typhoid outbreak prior to their relocation was exacerbated by flood water that spread through sites of open defecation around the village. Several people said that the old site was not healthy because it often flooded, with one resident explaining that ‘*down there it’s soggy and the place is waterlogged*’. Further, food security was compromised as coastal flooding led to saltwater intrusion that damaged subsistence crops such as breadfruit trees: ‘*when the water came up it was dirty, it brought mud into the village. Sometimes it affected the crops. It damaged the vegetables in our backyard gardens*’ (Man, 2020).

Nonetheless, health risks—specifically those linked to environmental changes—at the old site were not viewed as a primary push factor for relocation. The relocation to higher land was reported to be precipitated by the need to move away from a place where coastal flooding and erosion threatened land and infrastructure:

My house was so close to the shore, about three paces to the coastal barrier and then the sea. When the tide and big waves come in, my kitchen is flooded and my kitchen is attached to the house. The water almost reached up to my knees. (Woman, 2019)

The only problem is the climate change we going through now. Because that was a really good site. The impact of climate change was really fast. the water will come from the sea and the river. the village be covered with water. (Man, 2019)

The coast is eroding. We did some sort of coastal protection but were destroyed by the impact of the waves crashing onto the coastal area and the flooding river. A few houses near the sea were destroyed, trees and coconut trees. That was what we see and it was fast. (Woman, 2019)

### 3.2. Disrupted Place Attachment

The site of Vunidogoloa’s relocation is on *mataqali* (clan) land, and within walking distance to the old village site. This short-distance relocation has enabled continued place attachment as they remain within customary land, and many residents make regular trips to the old site for fishing and farming. Residents are happy that there is a viable future for their village and community, without ongoing direct exposure to flooding, coastal erosion and saltwater intrusion.

Yet, many residents said that they were sad to leave the old site: a place with which they had strong connections to land and ocean, and personal and ancestral connection. One older woman explained, for example, ‘*I was so sad to leave the old site*’ (Woman, 2019); even four years after their relocation, she misses their home, fishing, and the connection to the sea and tidal rhythms: ‘*We had good houses down there. It is hard then to be here. We do not know when it is high tide or low tide*’. Others highlighted the sense of rupture due to moving from a site with personally significant histories, distance from burial grounds, and loss of valued everyday activities such as fishing and swimming in the *qoliqoli* (customary fishing rights area):

Our forefathers were buried there, and my husband was buried there too. So I still feel that connection to there. I always go back to the burial ground. (Woman, 2016)

We remember the old site because a lot of us were born and raised there. And our parents had been raised there, plus we also have our ancestors buried down there. (Man, 2019)

When I go down to the old site then I get emotional as that was the place I first fell in love and married my husband. A good place. (Woman, 2019)

I’d rather stay near to the sea. To feed the children and all the family. Go swimming there. We went a lot, with the net. Four or five times a week. We had access to fish, shells. (Woman, 2020)

A few noted that the distance from the customary fishing area—*qoliqoli*—means that it is difficult to control and manage their coastal resources. One woman, for example, spoke of increased activity of poachers since they moved from the coast and have not been able to monitor use of their fishing grounds and coastline:

We lost our rights to our natural resources, especially our qoliqoli. When we moved up here anyone can just come and use our fishing grounds, poachers, and there’s no control. When we were down there we had control of how our resources are used. And also the chopping of the trees and the mangroves. So, we can’t safely keep our natural resources. All these things are happening at the old site because we are not there are the old site. (Woman, 2020)

Planned relocation, even while within customary land, affects place attachment as place-based things of value—fishing practices, monitoring and managing coastal resources, knowledge of tidal rhythms, proximity to sites of personal and community significance—have been disrupted and lost: these changes to place attachment affect the emotional health and well-being of some.

### 3.3. Food Security and Dietary Change

Food security has been enabled through continued, and even improved, access to customary farmland with fertile soil. The relocation site is closer to farmlands where people (mainly men) grow kava, cassava and other crops that they rely on both for subsistence foods and income. As one man explained, ‘*we’re nearer to the farm area here, we don’t need to walk a long distance. So everything has been basically easier to get to from here*’ (Man, 2019). Additionally, there is fertile soil such that subsistence kitchen gardens have readily been re-established by residents, with fruit trees and garden plots maintained throughout the village. Donor organisations also provided some assistance in diversifying agriculture and fishing practices, through the construction of trout farms and development of cash crop plantations including pineapples and bananas. Improved food security was noted as a key advantage of relocation. In 2015, one year after their relocation, one village leader explained:

The food security that we don’t have in the old site, we have that here. Vegetables, cash crops, we can’t plant that because of the sea water in the old site. Salinisation. Now we have pineapples, tapioca, dalo [taro], bananas. (Man, 2015).

However, village residents frequently commented on changes to their diet in the new site, noting reduced consumption of fresh seafood and increased consumption of processed foods. With the village situated 2 km inland, it is harder to access coastal fishing sites. Many people reported that they eat less fresh fish and seafood at the new site. Women frequently said they missed fishing, a key activity at the old site:

The old site is still better. Here nothing to do except weave and do the pandanus leaves for weaving. Down there we can go out to the sea go and fish. Here when there is nothing to cook then it’s nothing. Not like down there. You just cook the cassava and while it’s cooking you can go to the sea and go fish. You got something to eat. (Woman, 2019)

Additionally, their nutrient-rich traditional diet (e.g., root crops, fish, seafood, vegetables) is increasingly supplemented with processed and packaged foods. This is reported to be a result of increased proximity to roads and transport that link to nearby towns with their ready availability of store-bought packaged foods including tinned fish, bread, biscuits, rice, flour and noodles:

I think there’s more eating of packaged food from the store up here at the new site. Down there we are near the sea and we had fresh food from the sea. Up here I noticed especially with school children eating packaged noodles. (Woman, 2019)

At the old village we had fresh air coming from the sea, plenty of seafood easy to catch and eat; fresh. In just twenty minutes you catch then boil the fish. Here, plenty of times, if you don’t go to the sea you drink tea and eat tinned dish. (Man, 2020)

Many residents attributed emerging health problems at the new site to their altered diet and daily life; ‘*here we just get all the things from the market, and it makes the people get sick*’ (Woman, 2019). They spoke of altered diet leading to tooth decay and increases in non-communicable diseases or ‘NCDs’, specifically ‘diabetes’ or ‘sugar’ which is the local term for diabetes. While some people had developed diabetes while living at the old site —a problem they attributed to consumption of shop-bought foods such as sugar and flour—they said that it was more difficult to sustain a healthy traditional diet and manage their health in the relocation site. One older woman, for example, was diagnosed with diabetes while living at the old site and said ‘*diabetes might have been with me for sometimes, and my mother was diabetic too*’. However, she explained that her diabetes worsened following relocation because she ate foods that the doctor advised against: *‘the foods that the doctor told me not to eat, I am eating them like sugar, milk, oil, cassava*’. Four years after moving uphill she had her leg amputated due to complications from diabetes. She said, ‘*since I have amputation I was not able to do the things I love to do before like going fishing and do works like what we women do. I wanted to do those things again*’. Similarly, other residents suggested that complications from diabetes had increased at the new site:

At the old site, there was not much problem with the NCDs. There was access to fish, and there was not much access to processed food. But it’s more of a problem at the new site. Access to packaged foods. There were not many people who were sickly, with sugar, because of their access to fish. Good protein down there, fish and seafoods. Up here we eat processed foods; maggi noodles, tinned fish. At the old site, we ate fresh fish every day. But at this new site, sometimes only Sunday we eat fish. I preferred food at the old site. (Woman, 2020)

Our diet has changed. Here it is near to the town. From the old village, it was easy; we can get plenty of food from there, fish and crab. Here we get food from the market. Some of them they can’t eat those kinds of foods. Diabetes. Up here there are plenty of people with diabetes. At the old village, we stay healthy. Eat fish, fresh. Here we buy everything, we eat a lot from the stores in town. Diabetes, was maybe in the body but the sickness never showed. When we came here three of them had amputations; two women and one man. When we came here those amputations happened. (Woman, 2020)

### 3.4. Houses and Households

Housing is a determinant of health, and new and adequate housing—with appropriate materials, design, layout and size, and thermal protection—is an essential part of relocation. The housing design and village layout in Vunidogoloa’s resettlement was agreed in consultation with community leaders and members. Thirty timber-framed houses were built to identical specifications to ensure equality of housing resources, each consisting of an open plan room with an attached flush toilet with septic tank, shower receptacle, washing basin with faucet, and a small solar panel for electricity. While initial planning stated that all houses would be equipped with a kitchen, during a second phase of construction, this did not eventuate: kitchens have been constructed by residents from salvaged materials, and women cook with wood-fire.

Village leaders and residents decided that each married couple would have their own home, rather than a few families sharing one home as they did at the old site. Many residents (laughingly) suggested that the birth rate has increased since relocating, as couples have increased privacy in their homes. One young woman explained:

People are having more children now. Another one was just born. Another three are pregnant. It’s increasing. Maybe sixteen children have been born here, since we moved. That’s a good result. More children. (Woman, 2020).

Others indicated that the smaller household size has reduced everyday pressures, particularly cooking and cleaning, looking after children, and interpersonal dynamics. One older woman, a mother of six children, explained that she enjoys living in a smaller household: ‘*it’s a big change in my life, it’s more quiet, less food we need to buy to feed the family. When you live in a big family it’s a struggle. Additionally, sometimes we can fight if you live altogether. This is more peaceful. A bit private’ (Woman, 2020).*

However, the shift to single-family households is said to have reduced opportunities for interaction, communal activity and sharing of resources. The village still practices *solesolevaki*; communal collaboration where residents work together on projects such as planting root crops, cleaning the village, or building maintenance. Yet, many people miss the ‘communal lifestyle’ of extended households, with families sharing and helping each other, and indicated that people are now ‘pulling in different directions’:

Before I live with my family, and some other families. We live in one household at the old village. I like it more to live one house for one family. You can still help each other. When we live one family it’s good. (Woman, 2020)

One small group of women agreed that the shift to ‘individual homes’ from ‘very extended families’ means that the welfare of the individual family is increasingly prioritised over the community or ‘big family’; as one woman said, people are increasingly ‘*interested in the individual contribution for their own small family, not the big family*’. (Woman, 2020)

A few women said they had experienced increased family violence since moving from communal living structures to smaller household units where ‘*husband and wife live in one house*’, as they have lost the protection that communal living affords. During one discussion with a small group of women, one woman said, ‘*I go through a lot when he is drinking. He always abuses me hitting*’, with another replying ‘*I have that too; hitting and punching when he’s drinking*’. They agreed that this is a ‘*family problem*’ that has been amplified by living in smaller household units. 

### 3.5. Water and Sanitation

At the old site, while there was good access to water via a gravity-fed system from a spring-water tank, each household (of multiple families) had one pit toilet, and only a few had showers. Many residents recalled that toilets did not work effectively during flooding and high-tide, and there were too few available so people often ‘used the bushes’:

In the old site we had pit latrines. Septic tank is better. It is closer to the house. Septic tank we couldn’t have in the old site because it was wet, the ground was too wet. (Man, 2015)

Even when you dig holes the water comes up. That’s one of the reason we hardly use the toilets when they flush the thing doesn’t go down so that’s why they go to the river. It’s like the toilets are just there for decoration. (Woman, 2019)

Water and sanitation services at the new site are improved. All houses have a shower, a flushing toilet connected to a septic tank, a concrete sink with piped water for washing hands and dishes, and running spring water supplied by a gravity-fed system from a reservoir tank above the village. Nonetheless, water supply at the new site has intermittent problems with supply. There is no filter installed in the water tank, so the pipes get blocked during heavy rain. When this occurs, residents fetch water by bucket from a faucet linked to the water system that serviced the old site. Intermittent water supply is particularly problematic for women as they are primarily responsible for washing and cooking:

Water is a concern, particularly for women. Washing, cooking. When they launched the project, it was rushed. The water tank was not done properly. So we have problems. Sometimes we don’t have water. There is no filter, so it gets blocked with leaves and dirt. Normally during heavy rain we don’t have water maybe ten times a year. (Woman, 2020)

However, improved water, sanitation and hygiene has reportedly led to improvements in child health. The local nurse who provides health services for Vunidogoloa reported significant reductions in conjunctivitis and other skin and eye infections in Vunidogoloa, as children are now washing their hands and faces with clean water as well as treating infections with medicine. The nurse explained:

This year eye infection has decreased. So that’s a great effort from the community. Children mostly. They are washing hands. And when they are contacted with conjunctivitis, we treat it with tetracycline and they take the basin of water and put their face in. This year has been a great improvement. This year nil cases in Vunidogoloa. It’s a great improvement. (Man, 2020)

### 3.6. Geographies of Access

Relocation to the new site has increased proximity and access to the road, transport, services and urban centres. One benefit is improved access to health services, including the local health centre in a nearby village and hospitals in Labasa and Savusavu. A man in his seventies explained that he was the village nurse at the old coastal site; he had some medicine and equipment and could perform small operations such as stitching up wounds. However, given the distance from the road it was difficult for people with more serious problems to access health services, with unwell residents occasionally needing to take a raft to a neighbouring village to access road transport:

We had a bilibili [raft]. If we had a problem, we take the bilibili to the other village and call for the transport to come and pick up the person. If it was not so severe we walked up to catch the bus. But here we can just easily get to the bus. (Woman, 2020)

Many residents said they more regularly attend the health centre since their relocation closer to the road and bus service, and children’s immunisation coverage is improved. As one woman said:

We are closer to the road, whereas before we had to walk from the village to catch the bus. Now we can reach the social services more easily. The bus goes three times a day. Now I go about once a month, but when I lived at the old site I went only once a year. (Woman, 2015)

However, while the road brings access to essential services, it also brings increased exposure to new people, places and products. This is a change that has been noted since early in the relocation, with the sudden increase in access to transport and urban sites. As early as 2015, residents reported sociocultural changes associated with increased mobility of people between other places, including urban areas, and the village: as one man said, soon after their relocation, ‘*many people coming in and interfere with our customs and culture, and this never happened in the old village*’ (2015), and similarly an older woman recalled that there ‘*was less disturbance from outsiders*’ (2015) at the old coastal site. While all village residents were previously Methodist, increased engagement with new people and ‘outsiders’ has brought new religious faiths that a few households have adopted (e.g., Seventh Day Adventists). Many residents, both younger and older, describe ongoing changes in the years since their relocation to ways of living and to health behaviors including increased alcohol consumption and smoking:

We have a lot of issues now, like teenage pregnancy, smoking, drinking of alcohol. People are starting new behaviors, grab new lifestyle that we didn’t have before. And it’s getting bigger and we can’t control it. At the old site, we could control it. Here there is access to the town. There are more social problems. People drink outside. Sometimes people are already drunk and they come back to the community. So turagi [village head] can’t control that. (Woman, 2020)

### 3.7. Agency and Social Capital

Finally, traditional leadership structures were central to decision making and developing consensus among community members. As with other iTaukei villages, Vunidogoloa has interlinked governance structures: the village headman (Turaga ni Koro) who reports to the Fijian government via the Provincial Office; the traditional village leader (the chief); village-level committees; and the church (Methodist). Unlike many villages, however, Vunidogoloa has only one main *mataqali* (to which the Turaga ni Koro, Village Chief and head of the Village Development Committee belong—indeed they are brothers), such that tensions between *mataqali* did not emerge. Additionally, key decisions related to planned relocation were undertaken through *talanoa* at which clan members could speak. These decision-making structures were important, providing leadership and helping to resolve contentious aspects of relocation processes. As one village leader explained:

Many people have asked how we have been able to relocate so easily and I tell them it is because we only listen to one voice. We move when we are told to move and really in situations like these, it is important that you have people who are willing to listen and obey instructions. (Man, 2019).

Similarly, many community members explained that while village leaders made the decision to relocate, community members were consulted and ‘*contributed with ideas during the village meetings after they made the decision*’. However, some residents pointed out those who are not from the main clan were not involved in village *talanoa* and decision making. One woman—who married a resident of Vunidogoloa but comes from a different village and clan—explained:

We were not consulted. Only the clan was involved in the decision-making. We just went along with the decisions made by the others. Our contribution was not included, like the other clan members, when we were relocating from the old site. (Woman, 2020)

This indicates there were varying levels of agency and involvement in planned relocation decision making among Vunidogoloa’s residents, determined in large by traditional governance structures. 

Further, while the community relocated as a whole, and aimed to sustain the integrity of existing social and political structures and community life—e.g., governance structures including the village head and Chief, and village development committees continue to operate—many villagers reported some erosion of traditional hierarchies and values. There is said to be growing reluctance of younger residents to abide by the ‘village laws’ such as wearing a *sulu* (i.e., the national dress of Fiji, similar to a skirt) in the village, not drinking on Sunday, maintaining a traditional hairstyle (i.e., *buiniga*, the traditional hairstyle for women). As a village leader explained, while their way of life was previously very strong, some villagers are starting to object to village laws and traditions, such that village leaders need to ‘*provide counsel and remind them why protecting our way of life and our clan relationships is important*’. (Man, 2019). In Fiji, the Village By Laws aim to ensure traditional leadership is upheld including establishment of a Village Council, traditional protocol and custom is followed, village-level decisions are respected and observed in everyday village life, environmental standards are maintained, and communities live harmoniously and peacefully [23]. In Vunidogoloa, the Village By Laws govern matters including participation in the village council, establishment of village committees, appropriate dress, consumption of alcohol and kava, making excessive noise, and participation in communal labor (*solesolevaki*). Older residents said that, since their relocation, communal traditions (sharing food and resources) and respect for the Village By Laws appears to be eroding among the younger generation:

Everything is lost. In the old village we shared food, we caught plenty of fish. We have a little bit lost that. People stop sharing. Maybe in five or ten years everybody will look out for themselves, not sharing. Because plenty of people in the first year when we came up here, were still sharing. But not now. Times change. (Man, 2020)

Conversely, younger residents suggested that they have new-found freedoms and rights.

### 3.8. Health over Time in a Site of Planned Relocation

In sum, planned relocation of Vunidogoloa has resulted in both health opportunities and challenges. Key facilitators for good health include: movement away from some environmental risks to health, access to adequate drinking water and sanitation, consistent availability of food including through farms and kitchen gardens, diversification of livelihood opportunities (e.g., via trout farms and new crops), improved access to schools and health services, and appropriate housing design. This provides a strong foundation for health. However, over the five years since their relocation, village residents spoke of new and unanticipated risks to health and wellbeing: altered diets including increased consumption of packaged goods, increased health risk behaviors among some villagers including smoking and drinking, loss of respect for ‘village laws’ such as restrictions on the consumption of alcohol, a reported increase in family violence in some households due to loss of communal living, and the impacts of disrupted place attachment for mental wellbeing. While residents state that they are happy to have relocated to the higher site, many note declines in the health of the community since their move to higher ground and say, for example, ‘*we were more healthy down there than up here*’. This suggests that planned relocation has altered the social determinants of health in complex ways, bringing both health opportunities and risks. 

### 3.9. Limitations

First, the experiences of villagers in Vunidogoloa may not represent or convey larger trends or likely futures for people and populations who relocate in ‘similar’ places in Fiji or elsewhere. Planned relocation may be due to diverse environmental changes and impacts, can be of varying distances (e.g., short distance moves within or beyond customary land, long-distance relocation within national borders, and relocation to another country), and can include a whole community or only part. Additionally, planned relocation experience is shaped by local sociocultural, economic, political, demographic and geographic factors. Health outcomes of planned relocation will be closely determined by local contexts and places; as such, findings may not be directly generalisable to other contexts. 

Nonetheless, the findings may be informative for similar contexts, such as relocation of other small settlements that are exposed to environmental and climatic risks in small island developing states. Indeed, case studies—intensive and detailed examination of a single context—have been identified as particularly important for identifying climate adaptation risks and opportunities, and developing understanding of human environment interactions and emerging responses to climate change. Subsequent meta-analyses can integrate insights from multiple case studies to develop more generalised findings and identify policy significance at global, regional and national levels scales [24].

Second, participant recruitment was not randomised, but rather sought to be sensitive to local customs (e.g., the village head called residents to *talanoa*) and to include people with a diverse array of experience. Therefore, the views of people who were willing to participate in discussions are represented, potentially obscuring alternative experiences and accounts. 

Third, while the research was collected at four points in time, the changing level of trust and familiarity with village residents may have altered the nature of conversations; for example, conversations at later time-points brought more complex health concerns to the fore (e.g., problems with alcohol consumption, loss of respect for village laws, family violence) which may have been because these were emerging problems or because people felt more comfortable discussing such themes. This makes it challenging to draw any conclusions about the changing nature of health risks and opportunities over time. 

Fourth, the study is subject to recall bias, with people comparing their recollections of life at the old site with current experience. Fifth, the findings do not report diagnosed rates of illness, but rather residents accounts of changing determinants of health and health outcomes; causal connections may be more complex or different to those that villagers report (e.g., proximity to health centre may result in increased diagnoses of NCDs, rather than altered diet changing NCD).

Finally, all research and researchers have implicit values [15]. This research entailed close contact with village residents, and a certain sympathy for their positioning of planned relocation as a response to climate risks that have been created by the actions and emissions of people in larger and richer countries. We also recognise that the people we talked with know our research interests—particularly as we engaged with many participants at multiple time-points—and that their comments may have been shaped to reflect these interests. We have attempted to minimise bias through accurate representation of the meaning of people’s words.

## 4. Discussion

Small Islands Developing States (SIDS) from the Pacific region have highlighted the effects of the climate crisis on the lives and livelihoods of Pacific Islanders, and have called out the paucity of action on greenhouse emissions by the main global powers. The emerging health impacts of climate change in Pacific Islands include exposure to frequent and severe weather events, increased risk of vector-borne diseases such as dengue, and increased food insecurity through declining agricultural yield and reduced availability of seafood [25,26]. However, limited research in the Pacific Region focuses on complex pathways via which environmental and climatic change shape human health. 

Planned relocation away from a site of environmental risk entails movement to a new site, with diverse and dynamic health opportunities and risks. The health outcomes of planned relocation are not determined solely by reducing exposure to climatic and environmental biophysical risks (e.g., flooding, coastal erosion, saltwater intrusion). They are determined also by broader socio-political, ecological, economic and cultural contexts and processes. The experiences of residents of Vunidogoloa illustrate that planned relocation can fundamentally alter these social determinants of health. 

First, many of the determinants of health to which residents of Vunidogoloa refer are connected not only to the immediate process of planned relocation, but are mediated by wider processes of sociocultural change that operate at the local, national and global levels. (2) Some of the most widely cited risks to health—i.e., altered diet, increased consumption of alcohol and tobacco—were understood by Vunidogoloa’s residents to be the result of diminished traditional values and practices. Other research in the Pacific Islands has also linked the diminution of traditional practices and ways of living—which incorporate healthy diet, close community relationships, and physical exercise—to a decline in health [27]. This wider research indicates that the health of Pacific Islanders is eroded by profound shifts associated with ‘modernisation’: altered diet and physical activity, greater availability of alcohol, increased wage employment and participation in cash economies, and disrupted social networks [27]. Therefore, diminution of traditional practices is not particular to planned relocation; processes of modernisation are shaping the health of populations across the Pacific Islands, and indeed globally. However, in Vunidogoloa, the pace and scale of this sociocultural transition has been apparently amplified by the closer proximity of the relocated village to the road and urban centres, thereby contributing to some health risks. 

For example, the shift from traditional diet and food sources to a diet with more packaged foods was one of the most significant concerns. With their relocation away from the coast, residents of Vunidogoloa are experiencing declines in access to traditional coastal food sources, particularly seafood. Other studies of population relocation (e.g., in Mozambique, Viet Nam and Inner Mongolia) have also identified problems with altered foods and diet due for example to loss of access to farmland, grazing grounds, hunting and fishing grounds, and reduced access to wild foods and medicinal plants [28]. However, changes to diet are not only due to relocation away from traditional food sources (e.g., fishing grounds) but also to increased proximity to non-traditional sources of food via urban centres. The relocated village is connected into broader commercial food systems that extend throughout Fiji and beyond. Villagers report that this is contributing to increased risk of NCDs among residents, particularly diabetes. This dietary transition is occurring widely in Fiji and the Pacific Islands; approximately, 75 percent of deaths in the Pacific Islands region are attributable to NCDs, and Fiji has one of the highest rates in the world with NCDs accounting for more than 80 percent of deaths [29]. In the Pacific, NCDs are attributable in large part to an increasingly Western diet including consumption of imported processed food that are energy dense but with low nutritional value, and increasingly sedentary lifestyles [30]. For Vunidogoloa’s residents, planned relocation has sped up and amplified these wider processes of dietary change. 

Additionally, planned relocation has reportedly brought increased exposure to other commercial determinants of health, including the ‘unhealthy commodities’ of tobacco and alcohol companies [31]. Commercial determinants of health refer to unhealthy commodities, market forces and policy environments that promote these commodities, and global drivers that facilitate distribution and use of these commodities. The reported increase in tobacco and alcohol is said to be an outcome of increased mobility of people between the village and nearby urban centres, and access to roads and transport that can bring these products into the village from town. This is not just about ‘lifestyle’ choices. With relocation and increased connectivity to urban centres, residents of Vunidogoloa are more exposed to the products of market-driven economies and globalisation that contribute to ill health [32]. Planned relocation, then, in the context of environmental and climatic change, can operate as a ‘risk amplifier’ of wider threats to health—in this case, dietary change and increased consumption of alcohol and tobacco among some residents. 

Second, planned relocation has altered social structures and social capital. Social capital refers to the quantity and quality of social connections and includes cognitive dimension (e.g., residents’ perception of social relations, e.g., perceived norms of mutual help, attachment to community), and structural dimension (e.g., residents’ participation in social networks and activities). Numerous studies have documented the disruptions to social capital and patterns of social organisation that occur during process of community displacement and resettlement, such as resettlement following disaster [28,33]. Some suggest that social capital is preserved or even strengthened after resettlement, with other studies finding that resettlement and displacement disrupt and erode social capital [33]. In Vunidogoloa, there were significant efforts to replicate a familiar village layout and to sustain and draw strength from communal practices and traditional governance structures (e.g., village leaders and committees). Indeed, local communities, including in small island developing contexts, are understood as important agents of change with valuable social structures and social capital that can support adaptive capacity [34,35]. Additionally, it is important to note that while Indigenous communities are often presented as static and traditional entities that are exposed and vulnerable to the risks of the outside world, they are dynamic social structures with histories of mobility and social change [36]. Therefore, even while seeking to sustain familiar social relations and structures, they have capacity to adapt to social change. 

Nonetheless, planned relocation has contributed to social changes that are concerning to some residents. These changes involve shifting intergenerational relationships, altered social processes and practices, disruption to cultural heritage, new household structures, and changes to sense of place and community [37]. The concern among village elders and leaders is that important social values and traditions that promote community cohesion and wellbeing are being rapidly undermined, particularly as the younger generation can more readily engage with urban populations and places. Conversely, younger residents suggest that they have new-found freedoms and rights. The specific impact of these reported social changes on health and wellbeing is complex and methodologically difficult to unpack. One aspect of social change, however, that has reportedly had unanticipated impacts on health and wellbeing is the shift from multi-family to single-family households. This is a decision that apparently had widespread community support during the relocation planning phase. Additionally, many people—particularly women—appreciate the reduced pressures of cooking and cleaning in smaller households, and the increased privacy. However, a few women reported that family violence has increased in the more private spaces of single-family households. Gender-based violence is common for many women in Fiji, with the incidence of domestic violence higher in rural areas than urban areas [38]. Therefore, planned relocation appears to create, interact with and amplify changes to social structures and relationship, sometimes in unanticipated and adverse ways with consequences of health and wellbeing.

Third, in Fiji, there is an essential link between iTaukei people and their land, which is expressed in the concept of *vanua* (important throughout the Pacific). The Fijian scholar, Ravuvu, explains that *vanua* refers to: the land that people identify with, including the plants and animal life; sociocultural traditions, customs, beliefs and values; and other social institutions that maintain harmony, solidarity and prosperity [39,40]. The residents of Vunidogoloa have relocated within *mataqali* land, land with which they identify and to which they belong. However, *vanua* and place attachments are still disrupted. Place attachment has been variously defined [41]. Broadly, attachments to place refer to the emotional bonds that develop over time that arise from familiarity, a sense of belonging, place-based practices, and cognitive ties between people and their socio-physical environment [42]. There is a sense of loss and sadness at being relocated from a place of attachment.

Other researchers have also argued that disrupted place attachment due to loss of liveability is often attended by feelings of loss and grief [43]. For example, Willox et al. describe mourning expressed by Inuit for a changed land and ecosystem in the context of climate change [44]. Additionally, in their examination of how climate change affects the mental health and well-being of Inuit communities in Northern Canada and farming communities in rural Australia, Cunsolo and Ellis underscore the grief associated with environmental changes wrought upon places of belonging [43]. For residents of Vunidogoloa, there is a double-layered sadness associated with both the perceived environmental changes (e.g., coastal erosion, flooding, loss of coastal trees) as well as loss of place attachment engendered through their relocation (e.g., reduced proximity and connection to the ocean, to burial grounds, to the sea breeze, and to place). Climate change impacts mental health through complex pathways, including grief as people experience climate-related losses to ecosystems and landscapes [12,43,45]. Disruptions to place attachment are critical challenges of planned relocation and mobility, with potentially adverse consequences for health and well-being [8,46]. This is particularly the case for Indigenous communities with strong connections to place, connections that are entwined with ancestors, cultural identity, management of ecosystems, livelihoods, and place-based knowledges and cultures [47].

## 5. Conclusions

This paper has examined understandings and accounts of the health impacts of planned relocation from the perspective of residents of a relocated coastal village in Fiji, Vunidogoloa. The qualitative data collected over a five-year period look beyond the initial period of relocation, and illuminate complex and dynamic social determinants of health over time. Since their relocation to higher land, residents variously report both health opportunities and risks. The findings highlight that if planned relocation and retreat are to be implemented successfully, both in Fiji and globally, a greater focus is needed on the social determinants of health. Planned relocation and retreat must address not only the basic foundations for human health (e.g., clean drinking water, adequate sanitation, appropriate housing, access to health services), but also important social determinants of health including place attachment, social networks, food environments, and the impacts of broader processes of globalisation and change. These results highlight the need for context-specific planning and adaptation programs that understand and address diverse social determinants of health, and meaningful involvement of community members in ongoing decision making such that they can better act on and adapt to dynamic determinants of health.

## Figures and Tables

**Table 1:** Summary of health impacts of planned relocation, reported by participants.

Health Impact	Benefits	Risks
Environmental change	Moved away from flooding, erosion and saltwater intrusion which had some adverse health impacts: salinization of subsistence gardens; infectious disease due to floodwater.	
Place attachment and wellbeing	Reduced anxiety about flooding, coastal erosion and saltwater intrusion; sense of viable future.	Disruption to place attachment, including to fishing grounds, ocean, and burial grounds
Food security and nutrition	Closer to farmlands; increased crop production for income and food (pineapples, bananas, etc.).	Reduced access to fish and seafood, and increased consumption of packaged foods.
Houses and household	Improved quality of housing; increased birth rate (due to greater privacy).	Less communal culture due to separate homes for each family; Some reports of family violence
Water, sanitation, hygiene	Springwater piped to homes; improved sanitation: flush toilets with septic tank; improvements in hygiene and reduced skin/eye infection.	No adequate waste-water drainage around village.
Geographies of access	Increased access to health services via road and public transport; improved immunisation coverage.	Access to alcohol and tobacco from stores in nearby urban centre, Savusavu.
Agency and Social capital	Traditional social structures sustained, with village head, village Chief, and committee structures involved in decision making; ‘Village Laws’ are in place.	Increased exposure to ‘new faces’ and connectivity to urban sites bringing changes to culture and identity (religion, dress, hairstyle); ‘Village Laws’ are not upheld by all residents.
